# A character-strengths based coaching intervention to improve wellbeing of rural community health workers in Madhya Pradesh, India: Protocol for a single-blind randomized controlled trial

**DOI:** 10.1016/j.conctc.2024.101377

**Published:** 2024-09-27

**Authors:** Ameya P. Bondre, Azaz Khan, Abhishek Singh, Spriha Singh, Ritu Shrivastava, Narendra Verma, Aashish Ranjan, Jyotsna Agrawal, Seema Mehrotra, Rahul Shidhaye, Anant Bhan, John A. Naslund, Steve D. Hollon, Deepak Tugnawat

**Affiliations:** a Sangath, 106, Good Shepherd Colony, Kolar Road, Bhopal, Madhya Pradesh, 462042, India; b National Institute of Mental Health and Neurosciences, Hosur Road, Lakkasandra, Wilson Garden, Bengaluru, Karnataka, 560029, India; c Pravara Institute of Medical Sciences, Rahata, Ahmednagar, Maharashtra, 413736, India; d Department of Global Health and Social Medicine, Harvard Medical School, 25 Shattuck St, Boston, MA, 02115, United States; e Department of Psychology, Vanderbilt University, Nashville, TN, 37023, United States

**Keywords:** Character strengths, Community health workers, Work stress, Randomized controlled trial, Positive psychology

## Abstract

**Background:**

There is scarce knowledge on the use of structured positive psychology interventions for reducing work-stress and improving wellbeing of rural community health workers in India, particularly the Accredited Social Health Activists (ASHAs) who are village-level (resident women, incentivised) lay health workers. This trial will test the effectiveness of a ‘character-strengths’ based coaching intervention compared to routine supervision on wellbeing (‘authentic happiness’) of ASHAs.

**Methods:**

This protocol is for a single-blind, parallel group randomized controlled trial comparing the effectiveness of a five-day residential workshop focusing on the use of character-strengths and subsequent 8- to 10-week remote telephonic coaching (weekly) to individually support ASHAs to improve their wellbeing, against routine health system support. The arms are intervention added to routine ASHA supervision (weekly, by the ASHA supervisor), and routine supervision alone (control arm). The target sample comprises 330 rural ASHAs in Madhya Pradesh, India. The primary outcome of mean Authentic Happiness Inventory (AHI) scores will be compared between arms at 3-month follow-up. Secondary outcomes will include an assessment of ASHA's self-reported affect, self-efficacy, flourishing, burnout, motivation, physical health symptoms, quality of life, and routine work performance indicators, and the consequent patient-level outcomes [e.g., service satisfaction and depression remission rates after receiving brief psychological treatment by trained ASHAs]. We will also evaluate the costs of developing and delivering the intervention.

**Discussion:**

This trial will determine whether a character-strengths based coaching intervention is an effective and scalable approach for reducing work-stress and improving wellbeing of rural ASHAs in low-resource settings.

## Highlights

• This trial evaluates whether character-strengths based coaching can reduce work stress of rural frontline workers in India.
• This is a single-blind, parallel group randomized controlled trial.
• The intervention includes a residential workshop focusing on the use of character-strengths and subsequent telephonic coaching.
• The primary outcome of mean Authentic Happiness Inventory (AHI) score will be compared between arms at 3-month follow-up.
• This trial is an early effort in India to reduce work stress and improve wellbeing among rural (women) health workers.

## 1 Introduction

Community Health Workers (CHWs) in low-resource settings experience high levels of work-stress [1,2], while serving the communities they live in, and bridging their health needs [[3], [4], [5]] with formal health system outlets such as primary care clinics. Accredited Social Health Activists (ASHAs) in India are one such cadre and play a significant role under the National Health Mission (NHM) to strengthen the delivery of primary health services in rural communities [6]. In India, there is typically one ASHA (woman, resident) per village (for a population of ∼1000) who receives an initial 23-day training on linking community members to basic health services, providing basic first aid and supplies, and mobilizing the community around water, sanitation, nutrition, and health issues [7]. There are more than a million ASHAs selected and trained in India, making it one of the largest CHW programs in the world [7].

ASHAs are vulnerable to work-related stress, contributed by unequal workload distribution [8,9] and gender inequities within the health system [10]. Prior studies have reported low compensation relative to work demands, and dependence on incentives compared to other salaried personnel with whom they work (such as Auxiliary Nurse Midwives), in addition to delays in receiving performance-based incentives, poor institutional support, inadequate respect from seniors and peers [11], and limited voice to express their concerns being at the bottom of health system hierarchy [12]. On the other hand, patriarchal values embedded within households and the wider village community, family expectations around gendered domestic responsibilities, low socioeconomic status, poverty, and limited education contribute to individual-level stress [8,10].

While addressing these issues will need continued structural changes at multiple levels of the health system and the society at large, the potentially more immediate solutions aimed at promoting mental health and well-being of ASHAs are worth examining. There is a scarcity of studies addressing stress among CHWs in LMICs such as India [1], and prior work has generally focused on improving the ‘external’ factors such as monetary incentives, supervision strategies, task-allocation and flexibility, public recognition, and rewards, with inadequate attention to ‘intrinsic’ factors such as mental health, coping abilities, and wellbeing of ASHAs themselves [13].

In this respect, the personal attributes or strengths of an individual may influence the negotiation with work-stress. Cross-sectional studies indicate positive associations between specific ‘character-strengths’ (e.g., inquisitiveness or gratitude) and psychological well-being among the nursing cadres [14,15], who are similar to ASHAs in their job roles, with the mediating effects of social support and self-efficacy [15], or engagement in work [16]. Recent experimental evidence among nurses has also shown that interventions, combining the use of character‐strengths with mindfulness-based practices, have reported higher absolute scores of ‘hedonic’ (pleasure-attainment) and ‘eudaimonic’ (meaning and self-realisation) well‐being, than exclusively mindfulness-focused interventions [17].

Keeping rural ASHAs in mind, there is scarce knowledge of the use of such intervention models through experimental designs for improving wellbeing, and an absence of longitudinal studies and randomized controlled trials [18]. Further, the typical approach of ‘identify and use’ the strengths in challenging situations, adopted by studies in other global settings [19,20] (with more educated and privileged workplace employees in high-income regions) would need substantial contextual adaptation for CHWs such as ASHAs in India, as shown in our process of development of the proposed intervention [13].

### 1.1 Study objectives and hypothesis

The objective of this two-arm randomized controlled trial is to compare the effectiveness of a character-strengths based coaching intervention on wellbeing of ASHAs relative to routine health system supervision that ASHAs already receive.

**Fig. 1:**
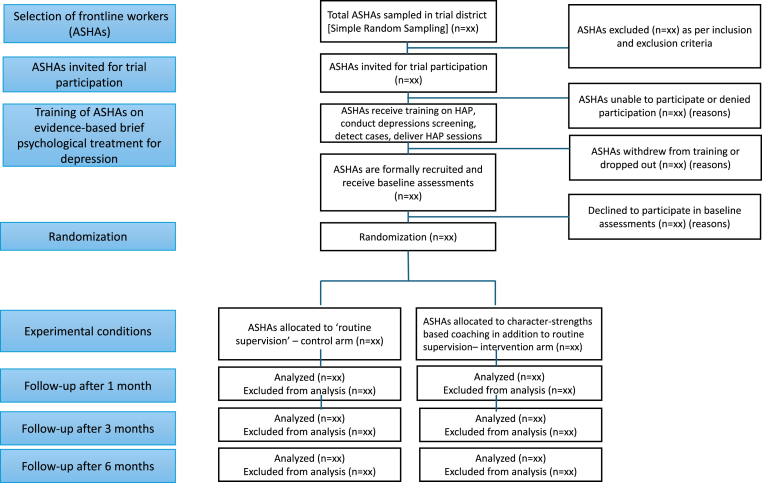
Trial CONSORT diagram.

The hypotheses include:

A) Primary hypothesis: ASHAs who receive the proposed coaching intervention (in addition to routine supervision) will show greater wellbeing than those who receive routine supervision alone.
B) Secondary hypotheses:
  a) ASHAs who receive the proposed coaching intervention (in addition to routine supervision) will show greater reduction in burnout and increase in motivation scores, than those who receive routine supervision alone.
  b) ASHAs who receive the proposed coaching intervention will show greater improvement in service delivery indicators (e.g., daily service contacts for routine services such as prenatal check-ups and conducting brief psychological treatment sessions for depression) than those who receive routine supervision alone.
  c) ASHAs who receive the proposed coaching intervention will show greater patient satisfaction for depression care services, and rates of remission in their patients' depression severity at endpoint, than ASHAs who receive routine supervision alone. (Note: All ASHAs prior to baseline assessment will be trained to deliver the brief psychological treatment for depression, see Fig. 1)

## 2 Methods

### 2.1 Study design

This study will have a two-arm single-blind parallel randomized controlled trial design with equal allocation of participants (ASHAs) between arms (Fig. 1). ASHAs in the control arm will be supported by their routine ‘ASHA facilitators or supervisors’ who typically supervise a group of ∼20 ASHAs on a weekly basis.

### 2.2 Study setting

This trial will be conducted in Sehore district in the state of Madhya Pradesh (MP) in central India. Being one of the largest states of India, MP has a population of over 72 million, nearly 73 % of whom live in rural areas [[21], [22], [23], [24], [25], [26], [27]] and it lags other Indian states with respect to human development and availability of resources [23,28]. About ∼1300 rural ASHAs work in Sehore district and as part of the study team's earlier trial, ∼330 ASHAs were trained to deliver an evidence-based brief psychological treatment for depression i.e., the Healthy Activity Program (HAP) [29].

### 2.3 Study procedures

#### 2.3.1 Participant recruitment

The trial will include Accredited Social Health Activists (ASHAs), working at the intersection of primary care and community levels, and currently not receiving any structured evidence-based coaching in improving their wellbeing and/or coping with work-stress [Note: We use the term ‘work-stress’ to indicate overall stress experienced by an ASHA, contributed by workplace, domestic (home/family environments) and village-level (her work in the community through house-to-house visits and village-level healthcare activities) stressors.]

Recruitment will start with the research coordinator and study data manager compiling a list of ASHAs from all blocks in Sehore district with the support of the District Program Manager, National Health Mission, and Block Medical Officers. This list will form the sampling frame for the trial. From the sampling frame, we will exclude ASHAs who have participated in mental health trainings, which could potentially equip them with mental health knowledge and techniques to manage their work-stress/burnout [30]. Finally, we will exclude ASHAs who have been a part of formative focus group discussions to develop the intervention for the present trial [31] and ASHAs who participated in the content testing phase of the intervention [13].

We have defined the ASHA selection criteria as follows:

*Inclusion criteria for ASHAs:* All rural ASHAs residing and working in Sehore district, Madhya Pradesh (verified in the health system records) who have been trained on delivering the evidence-based brief psychological treatment for depression [29] will be included in the trial. *Exclusion criteria for ASHAs:* a) ASHAs who plan to migrate within six months of recruitment and b) ASHAs who do not plan to continue working, or those who have resigned or planning to change jobs within six months of recruitment.

We have also defined the selection criteria for patients who will receive depression care by the ASHA, in the course of their routine work.

*Inclusion criteria for patients:* All persons identified by the ASHA in the community with a score of 10 or above on the nine-item Patient Health Questionnaire [32] who consent to participate in the trial. *Exclusion criteria for patients:* a) children and adolescents (10–19 years of age as defined by WHO), b) patients with significant speech, hearing, language or cognitive impairment, c) patients needing urgent medical or psychiatric attention, d) patients not planning to stay in the trial location for at least 3 months after initial screening, and e) patients not understanding Hindi.

#### 2.3.2 Participant training on the evidence-based brief psychological treatment for depression

In the sampling frame, we will sample a target of 330 ASHAs for orientation and training on the Healthy Activity Program (HAP), an evidence-based brief psychological treatment for community-based care for people identified with depression [29]. We will use simple random sampling (SRS) without replacement for this purpose. SRS will offer maximum sample diversity, equal chance of representation of all ASHAs, high internal and external validity and ease of data analysis [33,34]. This is about 25 % higher than the required number of ASHAs (n = 244, refer 2.6) to account for potential attrition/loss to follow-up and health workers who may decline to participate in HAP training and/or further study processes i.e., baseline assessments, intervention activities and outcome assessments (Fig. 1). The sampled ASHAs will be assigned unique participant IDs, which will be used to contact them regarding trial recruitment. These IDs will stay with the ASHAs throughout the trial period. The research team will call the sampled ASHAs from the list to invite them to participate in the study, starting with an in person orientation on the digital training platform [29] and training for delivering the HAP. After orientation and training completion, ASHAs will start community screening for depression and delivery of 6–8 HAP sessions (every ∼8–15 days) for each patient. ASHAs will also receive fortnightly peer group supervision for the delivery of HAP as documented in previous contextual studies [29,35]. A dedicated team of HAP supervisors, through the MindScroll Learning Management System [36], will supervise the ASHAs’ challenges in delivering HAP, and also retrieve the data on delivery of HAP sessions.

#### 2.3.3 Baseline assessment

ASHAs who have completed HAP training, delivered HAP sessions to patients, and received peer group supervision for their delivered HAP sessions, will be invited to come to their nearest community/primary health center to participate in a group information session. A trained research team member will explain the goals of the study to the group and what their participation will involve, depending on the (subsequent) arm allocation. We will provide an overview of the time required to participate in either of the experimental conditions (intervention or control arm) and the completion of study outcome assessments. ASHAs who are interested and willing to participate will meet with a research assistant, who will confirm eligibility and review a study information sheet and consent form with them. The research assistant will ensure that the ASHA has fully read and understood the participant information sheet and consent form before obtaining written informed consent. We will document the number of ASHAs who are eligible to participate, who decline participation (with reported reasons) and who are lost to follow-up.

After consenting, ASHAs will complete the baseline assessment. A research assistant will collect demographic information (including, for example, age, years of work experience, education level, and ownership of assets), and participants will self-report the primary outcome of wellbeing (i.e., authentic happiness), and additional measures (i.e., ASHA's affect, self-efficacy, flourish, burnout, motivation, physical health symptoms and quality of life). The baseline assessment will require approximately 2–3 h to complete. ASHAs will take short breaks at any time during the assessment and will be provided with snacks. The research assistant will be present throughout the process to respond to queries and ensure that ASHAs are able to complete the assessment forms.

From the standpoint of patient recruitment, all patients (of both arms) who score ≥10 on the nine-item Patient Health Questionnaire (PHQ-9) and have agreed to receive the first HAP session by the ASHA will be approached for participation in the study, and after obtaining written informed consent, the research team member will administer the baseline assessment (i.e., social and demographic information) to the patient. We will use the PHQ-9 score as assessed by the ASHA prior to starting HAP as the ‘baseline’, and the endline assessment including a repeat PHQ-9 and assessment of patient satisfaction, conducted by the research assistant after 3 months, will allow a comparison of pre- and post-HAP PHQ-9 scores, in addition to between-arm comparison of remission rates at follow-up.

#### 2.3.4 Randomization

Following the baseline assessment, ASHAs will be randomly allocated (1:1) to one of two study arms using Stata-17 [37]. Stata allows the researcher to set the ‘seed’ used to start the random number generator (RNG) process, so that it is reproducible; and it tracks the ‘state’ which the RNG is in before each command is run. Therefore, with each command, the RNG ‘state’ within the seed changes, which maintains the randomness of each subsequent command. Since we will train all ASHAs on HAP prior to randomization, any potential effects of mental health training on ASHAs' own wellbeing will be balanced between the arms. Prior to randomization, we will first stratify ASHAs who will complete baseline assessments using age (in years) and education level (number of total years of education), as these are some of the variables that may affect an ASHA's absorption of the intervention content and potentially affect the wellbeing outcome. Stratification will ensure that age- and education-distribution between the arms is balanced. Research team will inform all ASHAs (regardless of arm allocation) of the scheduled dates of their next (1-month) follow-up assessment, and so on for 3-month and 6-month follow-ups. Further, a separate research assistant will call the ASHAs and confirm their availability to attend the intervention residential workshop (if allocated to the intervention arm). ASHAs allocated to the intervention arm will receive the workshop within 7–10 days of baseline assessment.

#### 2.3.5 Interventions

##### 2.3.5.1 Routine health system support

As per National Health Mission guidelines [38], supervisors (‘facilitators’) provide support to ASHAs and ‘mentor’ them to improve their effectiveness, particularly for ASHAs who function poorly on many of their tasks or remain absent in periodic meetings/trainings. Possible reasons for poor functionality of these ASHAs include their low skill or knowledge levels, delays in payments, irregular medication supply for their services, inappropriate behaviours of health system staff, lack of family support for working, illness in the family, other social barriers, or no interest in working as ASHAs. Therefore, routine supervision will be treated as the standard of care in this trial. ASHAs in the intervention arm will receive the proposed positive psychological coaching intervention in addition to routine supervision. ASHA supervisors will not be involved in the coaching intervention activities i.e., residential workshop and remote coaching, due to hierarchical differences between supervisors and ASHAs, which can potentially affect ASHAs' reception/experiences of the intervention.

##### 2.3.5.2 Positive psychological coaching intervention added on to routine support

The proposed intervention will include a five days residential ‘coaching workshop’ for ASHAs, followed by ‘telephonic coaching support’ to enable ASHAs to use the learnings from the workshop to address stressful situations arising in the field during their routine work. As part of the design, we expect the residential workshop to be led by a facilitator (AK, also the intervention development lead) for batches of 15–20 ASHAs each, and the telephonic coaching to include phone calls (∼30–45 min in duration) between the assigned intervention coach and the ASHA, every week/every 10 days, lasting for 8–10 weeks. The trial team will include a dedicated group of eight intervention coaches for a required sample of 122 ASHAs who will be allocated to the intervention arm. The residential workshop will be conducted in the Sehore district team office, with staying and food arrangements for ASHAs. A pre- and post-workshop knowledge assessment will be conducted. A survey capturing the cost of workshop delivery and including the cost incurred by the ASHA due to the workshop will be administered to the workshop batch on day-5. The detailed process of intervention development, from a review of positive psychology and intervention development literature, to developing the intervention blueprint, and its contextual adaptation resulting into the modules of the workshop has been published earlier [13]. The process of intervention development generated a ‘Content Manual’ including four modules for handover to the ASHAs (for their reference throughout the trial, and beyond), and a detailed 5-day comprehensive plan for content delivery using a workshop ‘Facilitator's Manual’, and other additional facilitation-related supporting materials (e.g., exercise sheets, charts, energizers, and PowerPoint slides) [13].

Remote telephonic coaching will commence a week after the ASHAs have completed the workshop, returned home and resumed their daily work. Each weekly coaching call will be scheduled based on ASHA's convenient time/availability. The coach assigned to a batch of ASHAs will be present during their 5-day workshop and have prior knowledge of ASHAs (mutual rapport), which will help the coach-ASHA interaction during the call. ASHAs are expected to discuss their weekly workplace challenges with the coach during the call and receive support by the coach to address work-stress resulting from these challenges, based on the strategies learnt in the workshop.

Before starting each call, the coach will seek ASHA's verbal consent for call recording – this is to maintain fidelity using a self-rated checklist. The team has self-developed a remote coaching supervision checklist based on related therapist competence checklists [39] used in the South Asian context. The items of the checklist reflect the coaching protocol and the discussions during coach training. Eight coaches will be divided into two groups and each week, randomly selected audio recorded coaching call sessions (1–2 per week) will be distributed to each group of four coaches, along with supervisors, for rating. These sessions will be reviewed by the supervisors (AK, APB) and the checklists, self-rated by the coaches delivering the calls, and peer-rated by other coaches will be discussed with supportive feedback during weekly supervision sessions. Self-, peer- and supervisor-rating, combined with weekly supervision meetings with supportive feedback will enhance learning and up-skilling of the coaches. This process will be followed for an expected eight batches of intervention workshops.

During the 4th and 8th call, the coach will also administer a short survey capturing the cost of remote coaching delivery and any expenses incurred by the ASHA due to the remote coaching (refer 2.4.5). From a participant compensation standpoint, each ASHA will receive INR 900 and INR 800 for a total of five days of workshop and eight coaching calls, respectively (∼ USD 20).

### 2.4 Study measures

All questionnaires will be self-administered using paper forms overseen by research coordinators and research assistants blinded to participants’ arm allocation. All assessments will be targeted for completion within seven days of the due assessment date in each arm. For each of the baseline, 1-month, 3-month and 6-month follow-up visits, the ASHA will receive INR 300 (∼ USD 3.5) as participant compensation.

#### 2.4.1 Primary outcome (ASHA-level)

Authentic Happiness Inventory (AHI) is a subjective measure for the assessment of happiness [40]. We have used the term ‘wellbeing’ to represent this specific measure of happiness (including hedonic (pleasure attainment) and eudaimonic (meaning and self-realisation) wellbeing). AHI includes 24 sets of five statements [e.g., ranging from 1 (“I feel like a failure”) to 5 (“I feel I am extraordinarily successful”)] from which, the respondent chooses the statement that best describes her feelings in the past one week. AHI has been designed for monitoring upward changes in happiness [40] and has often been used in positive psychology intervention studies [[41], [42], [43], [44]]. Internal consistency at pre-test has been reported to be high (Cronbach's α = 0.94). AHI has been validated for construct validity, and linguistically/contextually adapted for this trial. AHI will be administered at baseline, 3 months (primary outcome assessment) and 6 months (potential long-term effects on wellbeing).

#### 2.4.2 Secondary outcomes (ASHA-level)

For assessing the possible explanatory variables, which could contribute to the hypothesized between-arm difference in wellbeing/authentic happiness scores at 3-month follow-up, the 20-item international Positive and Negative Affect Schedule (PANAS) Short Form (I-PANAS-SF) will be used to assess ASHA affect [45]; the ‘Flourish index’ (by VanderWeele, T.J.) including two questions from each of the five principal domains of happiness and life satisfaction, mental and physical health, meaning and purpose, character and virtue, and close social relationships, will be used to assess flourishing [46]; and the Occupational Self Efficacy Scale (OSES) will be used to assess self-efficacy [47]. These measures will be administered at baseline, 1- and 3-months. For assessing the levels of variables related to wellbeing/authentic happiness, the emotional exhaustion sub-scale of the Maslach Burnout Inventory–Human Service Survey [48], the motivation scale pretested for Indian frontline health workers [49], the 14-item Physical Health Questionnaire [50] to assess somatic symptoms (contributed by work-stress/burnout) and the health-related quality of life survey (EQ-5D) validated for the Indian population [51] will be used at baseline, 3- and 6-months.

Daily/weekly/monthly/quarterly service delivery measures (depending on the type of service and as routinely monitored by the health system) will represent a global measure of routine ASHA services (non-mental health) in terms of their contact points with the community, for example through home visits, facility contacts, and community visits. We will categorize this outcome as per the type of services, such as proportion of antenatal check-ups, vaccinations, or post-natal care visits completed out of the expected for a defined period (e.g., month or quarter, as gathered by the health system). We will collect the service delivery data from the district team from the period starting from baseline till 6-month follow-up for between arm comparisons. We will gather data on the fidelity of the routine supervision activities from ASHA supervisors of both arms as part of data collection for secondary ASHA-level outcomes to monitor the ‘standard of care’ as discussed in section 2.3.5.1 and Table-1. Besides this aspect, there is no involvement of the health system appointed ASHA-supervisors in the study or the intervention. Refer Table 2 for details on ASHA-related outcome measures.

**Table 1:** Routine Supervision and Character-strengths based coaching intervention.

	Routine Health System Supervision	Character-strengths based coaching intervention
Who delivers the support?	ASHA Facilitator or Supervisor	Intervention workshop facilitator (AK) and remote coaching support team
Who is the target of the support?	ASHAs (n = 244, or all enrolled ASHAs of both arms)	ASHAs (n = 122) of the intervention arm
Components of support	As per National Health Mission guidelines [38], supervisors (‘facilitators’) provide support to ASHAs and ‘mentor’ them to improve their effectiveness, particularly for ASHAs who function poorly on many of their tasks or remain absent in monthly meetings/trainings. Possible reasons for poor functionality of these ASHAs include their low skill or knowledge levels, delays in payments, irregular medication supply for their services, inappropriate behaviours of health system staff, lack of family support for working, illness in the family, other social barriers, or no interest in continuing as ASHAs. Therefore, routine supervision will be treated as the standard of care. Typically, supervisor meetings with ASHAs take place weekly, in person, in groups of ∼20 (1 supervisor for ∼20 ASHAs).	1. ASHAs will continue to receive routine supervisory support as discussed previously. 2. ASHAs will receive a five days residential ‘coaching workshop’ followed by remote individual ‘telephonic coaching support’ to enable them to use the learnings from the workshop to address stressful situations arising in the field during their routine work. As part of the design, we expect the residential workshop to be led by a facilitator (AK, also the intervention development lead) for batches of 15–20 ASHAs each, and the telephonic coaching to include phone calls (expected 30–45 min in duration) between the assigned intervention coach and the ASHA, every week/every 10 days, lasting for 8–10 weeks. The intervention team will include a dedicated group of eight coaches for a required sample of 122 ASHAs who will be allocated to the intervention arm. The residential workshop will be conducted in the Sehore district team office, with staying and food arrangements for ASHAs.

**Table 2:** ASHA-level outcome assessments.

Measure	Instrument	Description	Rationale for selection	Hypothesis	Timeline
**Primary Outcome**					
***Wellbeing***	Authentic Happiness Inventory (AHI)	Authentic Happiness Inventory (AHI) is a subjective measure for the assessment of happiness [40]. AHI includes 24 sets of five statements [e.g., ranging from 1 (“I feel like a failure”) to 5 (“I feel I am extraordinarily successful”)] from which, the respondent chooses the statement that best describes her feelings in the past one week. AHI has been designed for monitoring upward changes in happiness [40] and has often been used in positive psychology intervention studies [[41], [42], [43], [44]]. Internal consistency of 0.94 at pre-test has been reported.	We have used the term ‘wellbeing’ to represent this specific measure of happiness – AHI items represent both hedonic (pleasure attainment) and eudaimonic (meaning and self-realisation) wellbeing.	Mean AHI scores will be significantly greater in the intervention arm or among ASHAs receiving the character-strengths based coaching intervention in addition to routine supervision, compared to ASHAs receiving routine supervision alone (control arm)	Baseline, 3- and 6-month follow-up
**Secondary Outcomes**					
***Affect***	10-item international Positive and Negative Affect Schedule (PANAS) Short Form (I-PANAS-SF)	The I-PANAS is developed as a measure of the affective component of subjective wellbeing. The five positive affect (PA) states include: active, determined, attentive, inspired, and alert. The five negative affect (NA) states include: afraid, nervous, upset, hostile, and ashamed. Higher scores on both PA and NA items indicate the tendency to experience a positive and negative mood. Respondents are requested to rate the statement on a 5-point scale (not at all to extremely) by comparing themselves during the past 2 weeks with their ‘usual selves’.	I-PANAS-SF has acceptable psychometric properties such as cross-cultural stability and factorial invariance [70] and there is prior evidence of this measure being used to assess affect in other workplace employees in India [71]	Total PA and NA scores will be significantly greater and lesser, respectively, in the intervention arm relative to the control arm	Baseline, 1- and 3-month follow-up
***Flourishing***	Flourish and Secure Flourish Index (T. J. VanderWeele, T.J.)	The “Flourish” index (FI) consists of two questions or items from each of the following five principal domains: happiness and life satisfaction, mental and physical health, meaning and purpose, character and virtue, and close social relationships [46]. Each of the questions is assessed on a scale of 0–10. The FI score is obtained by adding the scores from each of these ten questions (total: 0–100). The “Secure Flourish” index (SFI) is similar to the FI, except for two additional questions on financial and material stability, which may indicate the capacity to sustain flourishing across the five principal domains. For interpretation, the FI and SFI scores are reported as averages of the questions (rather than sums), therefore all scores are scaled from 0 to 10.	The questions in FI are based on existing questions, which have received empirical validation and are widely used in studies on wellbeing. Studies have put forth the psychometric properties of both these indices [72] -evidence of validity and reliability (Cronbach's α = 0.89 for FI; Cronbach's α = 0.86 for SFI).	Mean FI and SFI scores will be significantly greater in the intervention arm relative to the control arm	Baseline, 1- and 3-month follow-up
***Self-Efficacy***	Occupational Self-Efficacy Scale	The scale consists of 19 items and uses a five-point Likert-scale with the response range varying from one for ‘strongly disagree’ and five for ‘strongly agree.’ Some sample items for this measure are ‘When confronted with a difficult task, I am willing to spend whatever it takes to accomplish it,’ and ‘I adjust quickly to challenges that come in my work.’	The scale's psychometric properties have been analysed in the Indian context [47].	Mean OSES scores will be significantly greater in the intervention arm relative to the control arm	Baseline, 1- and 3-month follow-up
***Burnout***	Emotional Exhaustion sub-scale of the Maslach Burnout Inventory–Human Service Survey	The overall questionnaire measures three burnout components: emotional exhaustion (8 items, Cronbach's α = 0.89), depersonalization (5 items, Cronbach's α = 0.69) and reduced personal accomplishment (7 items, Cronbach's α = 0.79). Emotional exhaustion is seen as the core component of this tool. Items are scored on a seven-point Likert's style scale – ‘never’ to ‘daily’ [48,57].	The Maslach Burnout Inventory (MBI) is used in most studies included in a systematic review on burnout in frontline primary health providers in low- and middle-income countries [1].	Mean MBI scores will be significantly lesser in the intervention arm relative to the control arm	Baseline, 3- and 6-month follow-up
***Motivation***	23-item structured motivation scale by Tripathy et al. (2016) for Indian rural health workers [49]	The tool has 23 items, with answers given on an agreement scale of 1–4 (1 = strong disagreement, 4 = strong agreement). Reverse coding is done for negative questions before analysis. The scale for negatively worded question is 1 (strong agreement) to 4 (strong disagreement). The tool has eight major constructs: general motivation, burnout, job satisfaction, intrinsic job satisfaction, organizational commitment, conscientiousness, timeliness and personal issues	The tool is adapted from a motivation construct developed by Mbindyo et al. which is adapted from Bennet et al. [49,73,74]. The tool has been used with community health workers in rural health facilities in India.	Mean motivation scores will be significantly greater in the intervention arm relative to the control arm	Baseline, 3- and 6-month follow-up
***Physical Health Synptoms***	14-item Physical Health Questionnaire (PHQ)	Respondents indicate how much they have been bothered by each of 14 symptoms in the past month. Response options are “not at all”, “bothered a little”, and “bothered a lot.”	Prior validation results showed that the PHQ is composed for four factors representing Gastrointestinal Problems, Headaches, Sleep Disturbances, and Respiratory Infections. The factors contributed to substantial amounts of item variance, and the PHQ dimensions demonstrated acceptable levels of internal consistency, except somewhat low for the Respiratory Infections subscale (0.66) [50].	Mean PHQ-14 scores will be significantly lesser in the intervention arm relative to the control arm	Baseline, 3- and 6-month follow-up
		*Somatic* symptom categories include gastrointestinal problems, headaches, sleep disturbances, and respiratory illness.			
		Average score is calculated for each participant.			
***Quality of Life***	EuroQol-5D and Visual Analogue Scale	The EQ-5D includes five questions on mobility, self-care, pain, usual activities, and psychological status with five possible answers for each item. The maximum score of 1 indicates the best health state, by contrast with the scores of individual questions, where higher scores indicate more severe or frequent problems. In addition, there is a visual analogue scale (VAS) to indicate the general health status with 100 indicating the best health status.	Norms for Indian population are available [51] based on a survey of 3548 adults in five states of India. The utility score was calculated using the EQ-5D-5L value set and norm scores were generated for age, sex, and other socio-demographic variables.	Mean EQ-5D and visual analogue scores will be significantly greater in the intervention arm relative to the control arm	Baseline, 3- and 6-month follow-up
***Service delivery measures***	Routine service delivery indicators as collected by the health system (e.g., related to antenatal checkups done, post-natal visits conducted, immunizations done)	A survey form will be generated with a list of routine ASHA performance indicators in consultation with the district health team. The form will include indicators for various services such as antenatal and postnatal care, reproductive health, and immunization. Examples of specific indicators include proportion of antenatal checkups expected out of total pregnant women in the ASHA's catchment area (for a fixed period e.g., a reporting month) and proportion of antenatal checkups completed out of total expected checkups.	Given the evidence-base, improved wellbeing can contribute to improved service delivery as reflected in changes in observed service delivery indicators [[65], [66], [67], [68], [69]].	Percentages of services delivered will be significantly higher in the intervention arm relative to the control arm	Data will be collected directly from the district health team for the period starting from baseline assessment to end of 6-month follow-up assessment.
		With respect to depression care, data on number of HAP sessions delivered, patients with completed treatment and patient dropouts will be gathered from ASHAs.			Number of HAP sessions delivered; number of patients with completed treatments; and number of patient dropouts will be compared between arms at 3-month and 6-month follow-up
***Cost of Intervention delivery***	Cost of ASHA's participation in the workshop (survey) and cost of participation in remote coaching (survey)	We will assess costs incurred by ASHAs due to their participation in their respective intervention workshop and remote coaching activities. A post-workshop questionnaire (on day-5) and a remote coaching questionnaire (during the 4th and 8th coaching call, covering data of the previous calls) will be developed and administered to capture individual-level costs incurred by ASHAs, such as items covering their workshop/remote coaching time, workshop travel time, workshop travel costs, smartphone data packs (remote coaching) and food/refreshment costs (residential workshop).	If the primary outcome results show significant differences in mean authentic happiness scores between arms at 3-month follow-up as seen in the pilot study (under peer review), estimating the cost of delivering such an intervention will have significance from the point of view of health system adoption.	Data only collected in the intervention arm	Post-workshop questionnaire administered to ASHAs on day-5 of each intervention workshop batch.
					Remote coaching questionnaire administered to ASHAs during the 4th and 8th coaching call, covering data of the previous calls.

With respect to depression care delivery, we will calculate the number of ASHAs’ HAP sessions, on a weekly basis. At 3-month and 6-month follow-up, we will compare the numbers of these HAP sessions between arms (Note: HAP treatment durations range from 6 weeks to 3 months), in addition to numbers of patients with closed (completed) treatments and drop-outs.

#### 2.4.3 Secondary patient outcomes

At baseline, we will administer the socio-demographic assessment (section-2.3.3) and retrieve the PHQ-9 score assessed by the ASHA prior to initiation of HAP. At 3-month follow-up, the PHQ-9 will be repeated to determine if the patient remitted. In addition, patient's satisfaction with HAP will be assessed using the Client Satisfaction Questionnaire (CSQ-8), used earlier in India with psychiatric outpatients [52,53]). Finally, we will administer the adverse event form to determine the reporting of any adverse/severe adverse events in the intervening 3-month period and report the data to Sangath Institutional Review Board (IRB). Refer Table 3 for patient-related outcome measures.

**Table 3:** Patient-level outcome assessments.

Measure	Instrument	Description	Rationale for selection	Hypothesis	Timeline
***Mental Health Service Satisfaction***	8-item Client Satisfaction Questionnaire	CSQ-8 is an eight-item tool to measure client's overall satisfaction with health services. Higher value of the composite score indicates more satisfaction [52].	A study by Tekkalaki et al. [53] in another Indian state used this tool to measure the satisfaction of psychiatric outpatients at a tertiary health center.	Mean patient satisfaction scores of patients receiving HAP will be higher in the intervention arm relative to the control arm.	3-month follow-up
***Remission or reduced depression severity***	9-item Patient Health Questionnaire (PHQ-9)	Patient Health Questionnaire (PHQ) 9-item assessment tool (Kroenke K et al. [32]) where occurrence of symptoms indicative of depression are asked to the patient to recall over a previous 2-week period, with each symptom severity ranging from 0 (not at all) to 3 (nearly every day).	Evidence for suitability of use for screening of depression in community mental health settings in India in view of acceptable psychometric properties [75].	We will assess the proportion of patients who show ‘early remission’ or attain a post-treatment score of <10 at 3-month follow-up [58] and compare these remission rates between the two arms. We hypothesize that patients linked to ASHAs in the intervention arm will show greater early remission rate than patients linked to ASHAs in the control arm	3-month follow-up

#### 2.4.4 Intervention process indicators

The intervention development team will monitor and document data on the number of ASHAs attending the batches of residential workshops, possible withdrawals during and after the workshop, and a pre- and post-workshop knowledge assessment. The average number of days between workshop closure and initiation of first remote coaching call will be calculated to assess the engagement of the ASHA. The number of coaching calls and average call duration will be recorded (minimum calls: 8). Further, the patterns of ASHAs re-scheduling the calls, with dropouts/losses to follow-up during the remote coaching phase will be documented.

#### 2.4.5 Cost of development and delivery of the intervention

From the point of view of development of the workshop and the remote intervention (blueprint, content development and content testing, as detailed elsewhere [13], and its pilot (October 2022 to May 2023), we will gather data from Sangath internal administrative team, based on similar prior studies involving development of training programs for ASHAs [54] such as information related to involved personnel (their roles, responsibilities, time spent, and salaries or payments), information technologies (e.g., smartphone data packs for remote coaching), travel (e.g., fuel expenses for content testing and pilot workshops), and infrastructure-related costs (e.g., vehicle, office space, workshop space, utilities).

As indicated in 2.3.5.2, we will assess costs incurred by ASHAs due to their participation in their respective intervention workshop and remote coaching activities. A post-workshop questionnaire (on day-5) and a remote coaching questionnaire (during the 4th and 8th coaching call, covering data of the previous calls) will be developed and administered to capture individual-level costs incurred by ASHAs, such as items covering their workshop/remote coaching time, workshop travel and travel costs, smartphone data packs (remote coaching) and food/refreshment costs (residential workshop).

#### 2.4.6 Post-trial focus group discussions

We will conduct focus group discussions (FGDs) with a purposive sub-sample of ASHAs in the intervention arm after the completion of 3-month follow-up assessments to understand their perspectives/experiences of the workshop and remote coaching intervention, and a second round of focus groups after 6-month follow-up assessments to explore any sustained (longer-term) application of intervention learnings by ASHAs to cope with work stress. FGDs will be moderated by a study team member who is not involved in intervention development/delivery or outcome assessments (RS).

### 2.5 Blinding

Research coordinators and research assistants administering study assessments to ASHAs and patients, will be blind to participant allocation, and study investigators (except data manager) will also be blind to participants’ experimental condition. Participants will be told their arm allocation only after consent and baseline assessment. There is complete separation between the team members involved in developing or delivering the intervention and assessing intervention fidelity and process indicator data (2.3.5.2 and 2.4.4), and the team members involved in administering the outcome assessments (2.4.1 to 2.4.3). Outcome team members and investigators will be unblinded after the completion of analysis of data of baseline, 1-, 3- and 6-month follow-up assessments.

### 2.6 Sample size estimation

In the absence of trials looking at the effectiveness of interventions aimed at improving wellbeing among community health workers in India, we referred to methodologically similar designs for calculating the sample size [[55], [56], [57]]. We will measure the change in wellbeing, pre- and post-intervention, in terms of mean happiness scores on the Authentic Happiness Inventory (AHI). We initially assumed a significant between-group difference of 0.19 points (effect size: 42 %) for the mean happiness scores at 3-month follow-up for the intervention arm (mean: 3.35, SD: 0.43) and control arm (mean: 3.16, SD: 0.48). With 90 % power and a two-sided α of 0.05, 122 ASHAs per arm are required to measure this difference by the independent group *t*-test.

The patient-outcome of depression severity will be measured by the nine-item Patient Health Questionnaire (PHQ-9) [32]. We will assess the proportion of patients who show ‘early remission’ or attain a post-treatment score of <10 [58] and compare these remission rates between the two arms at the patient's 3-month follow-up assessment. For the patient-outcome, we will refer the early remission rates in the PRIME Cohort study [58] due to its contextual similarity to our trial and assume rates of 40 % and 60 % in the control and intervention arm respectively. With a two-sided α of 0.05, and 80 % power, we will need 97 patients with depression per arm. Therefore, each of the 122 ASHAs per arm will be required to provide HAP sessions to at least 1 patient, accounting for dropouts from treatment and loss to follow-up.

### 2.7 Data management

Participant data and study assessments will be stored in REDCap [59] with date and timestamps for all data entry, and audit trail for subsequent changes. Data from paper/form assessments will be double entered into REDCap by (at least) two separate research assistants. After completion of data entry, the paper forms will be stored in a locked cabinet in a secure research office. Data checks will be performed at regular intervals throughout the trial period, and queries will be resolved immediately, while maintaining the audit trail. Intervention process indicators and fidelity data will be collected on paper and exported to a spreadsheet. Cost data on expenses incurred by ASHAs during the remote coaching phase will be collected through Google survey forms with backend analysis. All quantitative data will be anonymized and linked for analysis only by participants' study ID. Audio recordings of coaching calls (used for supervision meetings) will be stored in a secure, password-protected folder, and a separate file linking study IDs will be kept password-protected. All data will be maintained on a secure server at Sangath, India, accessible only to the core research team. The Sangath servers comply with the Health Insurance Portability and Accountability Act's (HIPAA) data security rules, required to operate the REDCap data management system. All study data will be de-identified (removed of personal identifiers), including those designated by HIPAA.

### 2.8 Statistical analysis

All outcome analyses will be intention-to-treat using imputation of missing outcome data by an appropriate method [60]. The primary outcome analysis will estimate the difference in the mean happiness score between ASHAs randomized to the coaching intervention versus routine supervision at baseline, and 3 and 6 months thereafter. We will assess moderation of coaching effect by *a priori* moderators (e.g., ASHA's age, education status and years of community experience) by fitting appropriate interaction terms and testing for heterogeneity of coaching effects in regression models. We will also assess the relationships between ASHA-level affect, self-efficacy and flourishing, and their more extrinsic outcomes such as authentic happiness, burnout, motivation, physical symptoms and quality of life, and the relationships between these extrinsic outcomes and their service delivery measures, patient satisfaction (of patients receiving depression care) and patient depression remission rates, by a combination of descriptive analyses, statistical tests of significance, and mediation analysis using the MacKinnon's approach [61]. From a patient outcome standpoint, we will compare the remission rates on PHQ-9 of patients between the arms at 3 month follow-up using the test of difference between two independent proportions.

During analysis of costing of intervention delivery, each item of expense will be examined as it relates to specific details about costs related to ASHA's participation in the workshop/remote coaching. Costs incurred by ASHAs over and above the monetary allowance provided by the study team for participation in the intervention workshop will be documented. Costs of participants' time will be measured by their average daily wage. The suite of measures will be based on those used for economic evaluation of the contextual ‘MANAS’ and ‘PREMIUM’ trials [62,63]. System-level costs required to deliver the workshop and remote coaching will be based on the World Health Organization (WHO) framework [64], which describes a health system in terms of six ‘building blocks’ - service delivery, health workforce, health information systems, essential medicines and technology, financing, and leadership and governance. The research team will gather cost information according to the relevant building blocks for the intervention arm. We will produce tables to show the total costs of the intervention arm and cost per participant in the arm (addition of system-level costs and average participant costs). Individual-level costs will be further broken down into direct costs (e.g., travel time and expenses) and indirect costs (e.g., extra working hours, or any pending health system activity [due to the participation in the intervention activities] that could have provided an incentive to the ASHA). System level costs will be broken down into the relevant building blocks.

### 2.9 Ethical considerations

Institutional Review Board (IRB) at Sangath, India has approved all study procedures. Written informed consent will be mandatory for enrolling ASHAs in this trial. Participation in the study is completely voluntary and ASHAs can decline participation or withdraw at any time without any consequence to their current employment as health workers. Health system officials will have no role in the consent process to minimize possible influence. Trial research assistants will be given periodic refresher training to observe their skills in administering consent and providing supportive feedback. Confidentiality of participants will be protected using unique study ID numbers, and by separating study data from participant-identifiable data. The research team will submit study progress reports, including serious adverse event (SAE) reporting, flow charts, and baseline characteristics of enrolled ASHAs and patients across the study arms to the IRB on a periodic basis. The team will maintain a file of all required regulatory documents, study-specific documents, IRB approval notices, IRB-approved consent documents, and all correspondence with the IRBs that will be available any time for study audit.

With respect to the temporary absence of intervention arm ASHAs from their village-level activities due to the 5-day residential workshop, we have duly considered the same. We want to highlight that we will provide 7–10 days for the ASHAs when telephonically inviting them in advance of their participation in the workshop, which will give them sufficient time to plan the activities in their village due to their absence. In addition, we will coordinate with the ASHA supervisor for the same, so her support is elicited in performing some of the health care duties in the village as needed. Further, a permission letter from the district-level Chief Medical and Health Officer (CMHO) will be obtained prior to inviting ASHAs for the residential workshop.

### 2.10 Trial management

The Senior Management Team (SMT), consisting of the principal investigator, co-investigators, and study collaborators, in addition to research and intervention coordinators, outcome evaluators, and data manager, will provide overall trial leadership and will meet at least every 7–14 days to review trial progress, participant recruitment, data collection, process indicators, and any safety concerns or other issues that may arise. The team will submit annual reports to the Sangath IRB, in addition to progress reports to the state health department.

## 3 Discussion

Our intended impact is to demonstrate improved wellbeing of frontline health workers by the delivery of a character-strengths based coaching intervention. The expected study results will position our team to propose this intervention model to be adopted by wider stakeholders. Importantly, these stakeholders include key cadres of frontline workers in India including ASHAs in other districts and states, Auxiliary Nurse Midwives who are health facility based, Aanganwadi Workers who work towards improving child nutrition and growth, as well as community health officers (CHOs) in sub-health centres. There are more than a million ASHAs in India (one of the largest community health worker programs in the world [7]) and this study would pave the way for addressing one of their most pressing challenges i.e., poor mental wellbeing and burnout. Given the evidence-base, improved wellbeing can contribute to improved service delivery with a consequent benefit to patients and communities [[65], [66], [67], [68], [69]]. Therefore, the trial is positioned to strengthen the overall primary care system by first improving the wellbeing and reducing burnout among ASHAs with the consequent improvement in delivery of their frontline services.

From the perspective of challenges at a broader level, we want to mention that post-pandemic, the government has also introduced training modules for ASHAs to make them aware of mental health conditions and coping with mental stress. While the ‘seeding’ of ASHAs by knowledge of mental health will affect all trial ASHAs, across both arms, we will consider gathering qualitative data on these aspects through the course of the trial, particularly during the focus group discussions (section 2.4.6). Another challenge could be the aspect of socially desirable response patterns in the answers provided to psychometric tools, especially given that ASHAs work at the bottom of system hierarchies. This will be addressed by standardised procedures of providing instructions to ASHAs during outcome assessment (e.g., emphasis on ‘no right and wrong answers’, standard operating procedures for outcome assessors to clarify ASHAs' queries, and providing a comfortable environment for outcome assessment).

We will disseminate our trial findings through conference presentations at leading international scientific meetings, as well as national meetings in India. From a scalability perspective, towards the end of the project, we will organize a 2-day Dissemination Workshop with relevant health system stakeholders and policymakers to share our findings and increase the uptake of evidence, particularly in the state government programs focusing on capacity-building, mental health and/or wellbeing of ASHAs.

### 3.1 Trial status

Enrolment for this trial started in January 2024 and is expected to complete by June 2024. Intervention activities are expected to complete by September 2024 and terminal follow-up outcome assessments by December 2024.

## 4 Conclusion

This trial will provide early evidence on the effectiveness of a character-strengths based coaching intervention to reduce work stress among rural frontline health workers in India, to inform similar interventions globally, particularly for the rural women cadres. Our study critically contributes to the broader positive psychology literature on the use of character-strengths for building capacities among frontline workers in low-resource settings to address/respond to work-stress.

## Funding

This work was supported by the 10.13039/501100011730Templeton World Charity Foundation, Nassau, Bahamas (Grant number: TWCF0635). The funding source had no role in the study design; collection, analysis and interpretation of data; writing of the report; and the decision to submit the article.

## Ethics approval

Institutional (ethics) Review Board at Sangath, India approved all study procedures.

## Consent

All participants provided written informed consent for the study activities.

## CRediT authorship contribution statement

**Ameya P. Bondre:** Writing – review & editing, Writing – original draft, Visualization, Validation, Supervision, Project administration, Methodology, Investigation, Funding acquisition, Formal analysis, Data curation, Conceptualization. **Azaz Khan:** Writing – review & editing, Visualization, Supervision, Resources, Project administration, Methodology, Investigation, Formal analysis, Data curation, Conceptualization. **Abhishek Singh:** Writing – review & editing, Visualization, Supervision, Resources, Project administration, Methodology, Investigation, Formal analysis, Data curation, Conceptualization. **Spriha Singh:** Writing – review & editing, Visualization, Supervision, Resources, Project administration, Investigation, Formal analysis, Data curation. **Ritu Shrivastava:** Writing – review & editing, Visualization, Supervision, Resources, Project administration, Methodology, Investigation, Formal analysis, Data curation, Conceptualization. **Narendra Verma:** Supervision, Resources, Project administration, Data curation. **Aashish Ranjan:** Writing – review & editing, Visualization, Validation, Supervision, Software, Methodology, Formal analysis, Data curation. **Jyotsna Agrawal:** Writing – review & editing, Writing – original draft, Supervision, Methodology, Conceptualization. **Seema Mehrotra:** Writing – review & editing, Writing – original draft, Supervision, Methodology, Conceptualization. **Rahul Shidhaye:** Writing – review & editing, Writing – original draft, Supervision, Methodology, Conceptualization. **Anant Bhan:** Writing – review & editing, Supervision, Project administration, Methodology, Funding acquisition, Conceptualization. **John A. Naslund:** Writing – review & editing, Writing – original draft, Supervision, Methodology, Conceptualization. **Steve D. Hollon:** Writing – review & editing, Writing – original draft, Supervision, Methodology, Conceptualization. **Deepak Tugnawat:** Writing – review & editing, Writing – original draft, Visualization, Supervision, Resources, Project administration, Methodology, Funding acquisition, Formal analysis, Conceptualization.

## Declaration of competing interest

The authors declare that they have no known competing financial interests or personal relationships that could have appeared to influence the work reported in this paper.

## Data Availability

Data will be made available on request.

## References

[1] Dugani S., Afari H., Hirschhorn L.R., Ratcliffe H., Veillard J., Martin G., Lagomarsino G., Basu L., Bitton A. (2018). Prevalence and factors associated with burnout among frontline primary health care providers in low-and middle-income countries: a systematic review. Gates Open Res..

[2] Pallas S.W., Minhas D., Pérez-Escamilla R., Taylor L., Curry L., Bradley E.H. (2013). Community health workers in low-and middle-income countries: what do we know about scaling up and sustainability?. Am. J. Publ. Health.

[3] Vasan A., Mabey D.C., Chaudhri S., Brown Epstein H.A., Lawn S.D. (2017). Support and performance improvement for primary health care workers in low-and middle-income countries: a scoping review of intervention design and methods. Health Pol. Plann..

[4] Scott K., Beckham S.W., Gross M., Pariyo G., Rao K.D., Cometto G., Perry H.B. (2018). What do we know about community-based health worker programs? A systematic review of existing reviews on community health workers. Hum. Resour. Health.

[5] Tulenko K., Mgedal S., Afzal M.M., Frymus D., Oshin A., Pate M., Zodpey S. (2013). Community health workers for universal health-care coverage: from fragmentation to synergy. Bull. World Health Organ..

[6] Mission N.R.H. (2005). National rural health mission (2005-2012)--mission document. Indian J. Publ. Health.

[7] Scott K., George A.S., Ved R.R. (2019). Taking stock of 10 years of published research on the ASHA programme: examining India's national community health worker programme from a health systems perspective. Health Res. Pol. Syst..

[8] Manjunath U., Sarala R., Rajendra D., Deepashree M.R., Chokshi M., Mokashi T., N M.S. (2022). Assessment of workload of ASHAs: a multi-stakeholder perspective study for task-sharing and task-shifting. J. Health Manag..

[9] Meena S., Rathore M., Kumawat P., Singh A. (2020). Challenges faced by ASHAs during their field works: a cross sectional observational study in rural area of Jaipur, Rajasthan. Int. J. Med. Publ. Health.

[10] Ved R., Scott K., Gupta G., Ummer O., Singh S., Srivastava A., George A.S. (2019). How are gender inequalities facing India's one million ASHAs being addressed? Policy origins and adaptations for the world's largest all-female community health worker programme. Hum. Resour. Health.

[11] Scott K., Shanker S. (2010). Tying their hands? Institutional obstacles to the success of the ASHA community health worker programme in rural north India. AIDS Care.

[12] Mishra A. (2014). ‘Trust and teamwork matter’: community health workers' experiences in integrated service delivery in India. Global Publ. Health.

[13] Khan A., Sharma L., Agrawal S., Nayak S.R., Shrivastava R., Ahuja R., Bondre A.P. (2023). Development of a character-strengths based coaching program for rural community health workers to address their work stress in Madhya Pradesh, India. Curr. Psychol..

[14] Burke R.J., Ng E.S., Fiksenbaum L. (2009). Virtues, work satisfactions and psychological wellbeing among nurses. Int. J. Workplace Health Manag..

[15] Xie J., Liu M., Zhong Z., Zhang Q., Zhou J., Wang L., Cheng A.S. (2020). Relationships among character strengths, self-efficacy, social support, depression, and psychological well-being of hospital nurses. Asian Nurs. Res..

[16] Ding X., Kan H., Chu X., Sun C., Ruan F. (2022). A study of character strengths, work engagement and subjective well-being in Chinese registered nurses. Med. Pr..

[17] Monzani L., Escartín J., Ceja L., Bakker A.B. (2021). Blending mindfulness practices and character strengths increases employee well‐being: a second‐order meta‐analysis and a follow‐up field experiment. Hum. Resour. Manag. J..

[18] Ghosh A., Deb A. (2016). Positive psychology progress in India: accomplishments and pathways ahead. Psychol. Stud..

[19] Harzer C., Ruch W. (2012). When the job is a calling: the role of applying one's signature strengths at work. J. Posit. Psychol..

[20] Pang D., Ruch W. (2019). Fusing character strengths and mindfulness interventions: benefits for job satisfaction and performance. J. Occup. Health Psychol..

[21] Sai Shankar P. (2015). A bird's eye view of census 2011. CVR J. Sci. Technol..

[22] Suryanarayana M.H., Agrawal A., Prabhu K.S. (2016). Inequality-adjusted human development index: states in India. Indian J.Hum. Dev..

[23] Menon P., Deolalikar A.B., Bhaskar A. (2009). https://www.ifpri.org/publication/comparisons-hunger-across-states.

[24] Shidhaye R., Shrivastava S., Murhar V., Samudre S., Ahuja S., Ramaswamy R., Patel V. (2016). Development and piloting of a plan for integrating mental health in primary care in Sehore district, Madhya Pradesh, India. Br. J. Psychiatr..

[25] Murthy R.S. (2017). National mental health survey of India 2015-2016. Indian J. Psychiatr..

[26] Shidhaye R., Lyngdoh T., Murhar V., Samudre S., Krafft T. (2017). Predictors, help-seeking behaviour and treatment coverage for depression in adults in Sehore district, India. BJPsych open.

[27] National Health Mission (2018). Nation health mission (M.P.). India. https://www.nhmmp.gov.in/.

[28] Suryanarayana M.H., Agrawal A., Prabhu K.S. (2016). Inequality-adjusted human development index: states in India. Indian J.Hum. Dev..

[29] Naslund J.A., Tugnawat D., Anand A., Cooper Z., Dimidjian S., Fairburn C.G., Patel V. (2021). Digital training for non-specialist health workers to deliver a brief psychological treatment for depression in India: protocol for a three-arm randomized controlled trial. Contemp. Clin. Trials.

[30] Naslund J.A., Tyagi V., Khan A., Siddiqui S., Kakra Abhilashi M., Dhurve P., Bhan A. (2022). Schizophrenia assessment, referral and awareness training for health auxiliaries (SARATHA): protocol for a mixed-methods pilot study in rural India. Int. J. Environ. Res. Publ. Health.

[31] Shrivastava R., Sharma L., Jolly M., Ahuja R., Sharma R., Naslund J.A., Bondre A.P. (2023). “We are everyone's ASHAs but who's there for us?” a qualitative exploration of perceptions of work stress and coping among rural frontline workers in Madhya Pradesh, India. Soc. Sci. Med..

[32] Kroenke K., Spitzer R.L., Williams J.B. (2001). The PHQ-9: validity of a brief depression severity measure. J. Gen. Intern. Med..

[33] Acharya A.S., Prakash A., Saxena P., Nigam A. (2013). Sampling: why and how of it. Indian J.Med. Spec..

[34] Bhardwaj P. (2019). Types of sampling in research. J. Prim.Care Specialties.

[35] Patel V., Naslund J.A., Wood S., Patel A., Chauvin J.J., Agrawal R., Fairburn C.G. (2022). EMPOWER: toward the global dissemination of psychosocial interventions. Focus.

[36] MindScroll. Home. (n.d.) Retrieved March 18, 2024, from https://mindscroll.com/.

[37] StataCorp (2017).

[38] National Health Mission. Handbook for ASHA facilitators. (n.d.). Retrieved March 28, 2021, from http://nhm.gov.in/images/pdf/communitisation/asha/Handbook_for_ASHA_Facilitators.pdf.

[39] Kohrt B.A., Jordans M.J., Rai S., Shrestha P., Luitel N.P., Ramaiya M.K., Patel V. (2015). Therapist competence in global mental health: development of the ENhancing Assessment of Common Therapeutic factors (ENACT) rating scale. Behav. Res. Ther..

[40] Seligman M.E., Steen T.A., Park N., Peterson C. (2005). Positive psychology progress: empirical validation of interventions. Am. Psychol..

[41] Ruch W., Proyer R.T., Harzer C., Park N., Peterson C., Seligman M.E. (2010). Values in action inventory of strengths (VIA-IS). J. Indiv. Differ..

[42] Schiffrin H.H., Nelson S.K. (2010). Stressed and happy? Investigating the relationship between happiness and perceived stress. J. Happiness Stud..

[43] Mongrain M., Anselmo-Matthews T. (2012). Do positive psychology exercises work? A replication of Seligman et al.%28%29. J. Clin. Psychol..

[44] Proyer R.T., Gander F., Wellenzohn S., Ruch W. (2014). Positive psychology interventions in people aged 50–79 years: long-term effects of placebo-controlled online interventions on well-being and depression. Aging Ment. Health.

[45] Thompson E.R. (2007). Development and validation of an internationally reliable short-form of the positive and negative affect schedule (PANAS). J. Cross Cult. Psychol..

[46] VanderWeele T.J. (2017). On the promotion of human flourishing. Proc. Natl. Acad. Sci. USA.

[47] Chaudhary R. (2014). Occupational self efficacy expectations among Indian executives: examining the psychometric properties of occupational self efficacy scale (OSES). Global Bus. Rev..

[48] Maslach C., Jackson S.E. (1986).

[49] Tripathy J.P., Goel S., Kumar A.M. (2016). Measuring and understanding motivation among community health workers in rural health facilities in India-a mixed method study. BMC Health Serv. Res..

[50] Schat A.C., Kelloway E.K., Desmarais S. (2005). The Physical Health Questionnaire (PHQ): construct validation of a self-report scale of somatic symptoms. J. Occup. Health Psychol..

[51] Jyani G., Prinja S., Garg B., Kaur M., Grover S., Sharma A., Goyal A. (2023). Health-related quality of life among Indian population: the EQ-5D population norms for India. J. Global Health.

[52] Attkisson C.C., Zwick R. (1982). PsycTESTS Dataset.

[53] Tekkalaki B., Tripathi A., Arya A., Nischal A. (2017). A descriptive study of pattern of psychiatric referrals and effect of psychiatric intervention in consultation-liaison set up in a tertiary care center. Indian J.Soc.Psychiatry.

[54] Joshi U., Naslund J.A., Anand A., Tugnawat D., Vishwakarma R., Bhan A., Lu C. (2022). Assessing costs of developing a digital program for training community health workers to deliver treatment for depression: a case study in rural India. Psychiatr. Res..

[55] Proyer R.T., Gander F., Wellenzohn S., Ruch W. (2015). Strengths-based positive psychology interventions: a randomized placebo-controlled online trial on long-term effects for a signature strengths-vs. a lesser strengths-intervention. Front. Psychol..

[56] Proyer R.T., Gander F., Wellenzohn S., Ruch W. (2016). Addressing the role of personality, ability, and positive and negative affect in positive psychology interventions: findings from a randomized intervention based on the authentic happiness theory and extensions. J. Posit. Psychol..

[57] Verweij H., van Ravesteijn H., van Hooff M.L., Lagro-Janssen A.L., Speckens A.E. (2018). Mindfulness-based stress reduction for residents: a randomized controlled trial. J. Gen. Intern. Med..

[58] Shidhaye R., Baron E., Murhar V., Rathod S., Khan A., Singh A., Patel V. (2019). Community, facility and individual level impact of integrating mental health screening and treatment into the primary healthcare system in Sehore district, Madhya Pradesh, India. BMJ Glob. Health.

[59] REDCap Consortium (2015, July 8). REDCap: research electronic data capture. https://www.project-redcap.org/.

[60] White I.R., Thompson S.G. (2005). Adjusting for partially missing baseline measurements in randomized trials. Stat. Med..

[61] MacKinnon D.P., Fairchild A.J., Fritz M.S. (2007). Mediation analysis. Annu. Rev. Psychol..

[62] Patel V., Weiss H.A., Chowdhary N., Naik S., Pednekar S., Chatterjee S., Kirkwood B.R. (2011). Lay health worker led intervention for depressive and anxiety disorders in India: impact on clinical and disability outcomes over 12 months. Br. J. Psychiatr..

[63] Buttorff C., Hock R.S., Weiss H.A., Naik S., Araya R., Kirkwood B.R., Patel V. (2012). Economic evaluation of a task-shifting intervention for common mental disorders in India. Bull. World Health Organ..

[64] Indicators A.H.O. (2010). https://iris.who.int/bitstream/handle/10665/258734/9789241564052-eng.pdf.

[65] Willis-Shattuck M., Bidwell P., Thomas S., Wyness L., Blaauw D., Ditlopo P. (2008). Motivation and retention of health workers in developing countries: a systematic review. BMC Health Serv. Res..

[66] Mondal N., Murhekar M.V. (2018). Factors associated with low performance of accredited social health activist (ASHA) regarding maternal care in Howrah district, West Bengal, 2015–‘16: an unmatched case control study. Clin. Epidemiol.Global Health.

[67] Dholakia R.H., Bajpai N. (2011). Improving the performance of accredited social health activists in India.

[68] Bhattacharyya K., Winch P., LeBan K., Tien M. (2001). Arlington: USAID BASICS II.

[69] Singh D., Negin J., Otim M., Orach C.G., Cumming R. (2015). The effect of payment and incentives on motivation and focus of community health workers: five case studies from low-and middle-income countries. Hum. Resour. Health.

[70] Thompson E.R. (2007). Development and validation of an internationally reliable short-form of the positive and negative affect schedule (PANAS). J. Cross Cult. Psychol..

[71] Dahiya R., Rangnekar S. (2019). Validation of the positive and negative affect schedule (PANAS) among employees in Indian manufacturing and service sector organisations. Ind. Commerc. Train..

[72] Weziak-Bialowolska D., McNeely E., VanderWeele T.J. (2019). Flourish index and secure flourish index–validation in workplace settings. Cogent Psychol..

[73] Bennett S., Franco L.M., Kanfer R., Stubblebine P. (1999).

[74] Mbindyo P.M., Blaauw D., Gilson L., English M. (2009). Developing a tool to measure health worker motivation in district hospitals in Kenya. Hum. Resour. Health.

[75] De Man J., Absetz P., Sathish T., Desloge A., Johnson L., Thankappan K.R., Williams E.D. (2021). Are the PHQ-9 and GAD-7 suitable for use in India? A psychometric analysis. Front. Psychol..

