# Supplementary material for: A character-strengths based coaching intervention to improve wellbeing of rural community health workers in Madhya Pradesh, India: Protocol for a single-blind randomized controlled trial
Source: Contemp Clin Trials Commun. 2024 Sep 27;42:101377. doi: 10.1016/j.conctc.2024.101377 (PMC11488449; doi:10.1016/j.conctc.2024.101377)
Supplement: Multimedia component 1 [file mmc1.docx]

**AANAND Intervention Blueprint (draft) v.01**

| **Module #** | **Module**  **Name** | **Topics/Problems Identified**  **(From prior focus groups with ASHAs)** | **Learning Objectives** | **Relevant Character Strengths to be used in Strategies**  **(Identified from prior strengths assessment of ASHAs)** | **Strategies/**  **Approaches** | **Activities** | **Materials** | **Outcomes** |
| --- | --- | --- | --- | --- | --- | --- | --- | --- |
| 1 | Welcome/  Introduction to CS | 1. Introduction to C.S.  2. Identification of key work problems  3. Strengths use for problems | 1. Understand the concept of C.S.  2. Identify top strengths (in lay terms)  3. Know the 24 C.S./6 virtues  4. Identify key problems in work  5. Recognize the use of CS for work problems | Self-expressed CS as part of introduction in lay terms (non-VIA terms) | NA | 1.Icebreaker, expectations from workshop  2. Myths and Disclaimers  3. Didactic  4.Discussion  5.Reflection  6.Brainstorm/Reflect on scenarios  7.Response to scenarios | 1.PowerPoint presentation  2. Pen and Paper  3. Reference material for content^1-2^ | Orient to the possible ways of using strengths in problem-situations at work, home and in the community. |
| References:  1. Foundations of Strengths Based Practice. Seven Core Concepts of the Science of Character. Chapter 1. Niemiec RM. Character strengths interventions: A field guide for practitioners. Hogrefe Publishing; 2017 Jun 30.  2. Harzer, C., & Ruch, W. (2012). When the job is a calling: The role of applying one’s signature strengths at work. Journal of Positive Psychology, 7(5), 362–371. https://doi.org/10.1080/ 17439760.2012.702784. | | | | | | | | |
| 2 | Responding to work problems | 1.Lack of acknowledgement (e.g., views, effort), and mistrust by supervisors  2.Staff Misbehavior  3.Lack of consideration for her personal factors (e.g., leaves, health, family related) | 1. Understanding the situation and your reaction  2. Differentiating what can be changed and cannot be changed  3. Addressing the issue based on #2 | 1.Wisdom  2.Self-Compassion, Self-discipline  3. Modesty and Humbleness  4. Courage  Also: Hope, Persistence, Social Skillfulness | 1.Evaluate the whole situation and ‘role’ of self  2. ‘Components of *Karma*^3^*’*  3. Develop personal strength-habits  ‘Strengths-Habit’ exercise^5-6^ [cue, routine, reward]  4. Emotional regulation^4^ 5. Inter-dependence with CHWs (induction module) | 1. Didactic  2. Roleplays (passive, assertive, aggressive) 3. Reflection  4. Scenario-based Discussion  5. Mindfulness activities (easy to use) -> breath focused e.g., 5-4-3-2-1 | 1. Scripts  2. Guides  3. Videos on Mindfulness | Learn the ways to address stress resulting out of poor/adverse staff/peer behaviors |
| 3. Dalal AK, Misra G. The core and context of Indian psychology. Psychology and developing societies. 2010 Mar;22(1):121-55.  4. Choubisa R, Singh K. Development and validation of a web-delivered positive psychological intervention in an Indian Milieu: Lessons from a limited pilot randomized controlled trial. Cogent Psychology. 2018 Dec 31;5(1):1488512.  5. McQuaid, M., & VIA Institute on Character (2015). VIA character strengths at work [Web log post]. Retrieved from <https://www.viacharacter.org/blog/category/via-character-strengths-in-use/>  6. Create a Stengths habit- with McQuaid,M, Available: <https://leadthroughstrengths.com/strengths-habit/>, Accessed: 26 March, 2022  7. Kabat-Zinn J. Mindfulness-based stress reduction (MBSR). Constructivism in the Human Sciences. 2003 Jul 1;8(2):73.  8. Monzani L, Escartín J, Ceja L, Bakker AB. Blending mindfulness practices and character strengths increases employee well‐being: A second‐order meta‐analysis and a follow‐up field experiment. Human Resource Management Journal. 2021 Nov;31 (4):1025-62. | | | | | | | | |
| 3. | Dealing with social issues (village/community-level problems) | 1.Discouragement and less support by village community  2. Prejudice (gender and caste) and Discriminatory behavior  3. Lack of acceptance of her role | Identify the strategies to respond to challenges posed by community members in the course of routine work | 1.Social Responsibility  2.Courage  3.Wisdom  4.Forgiveness  5. Practical and far-sightedness  6. Social skillfulness  7. Persistence  8. Hope  9. Leadership  10. Love | 1.Build on the learnings of the last module  2. Greater good -Asakti(attachment)-Anasakti (non-attachment); Mind v/s Intellect^3^’^11^ e.g., What matters most, Defining moments  3. Stories: change makers/senior ASHAs  4. Appreciating resources, support (Compassion-self and others)  5. Specific: Implementation Intention^9-10^  Negotiation/Communication  Mindfulness | 1.Cases and Breakout groups  2. Roleplays  3. Mindfulness activities  4. Appreciative inquiry (positive moments) | 1. Case descriptions and prompts  2. Scripts  3. Videos on Mindfulness | Learn to negotiate with existing levels of community acceptance and prejudice to continue work |
| 9. Hudson, N. W., & Fraley, R. C. (2015). Volitional personality trait change: Can people choose to change their personality traits? Journal of Personality and Social Psychology, 109(3), 490–507  10. Dalton, A. N., & Spiller, S. A. (2012). Too much of a good thing: The benefits of implementation intentions depend on the number of goals. Journal of Consumer Research, 39(3), 600–614.  11. Pandey J, Singh M. Asakti-Anasakti as mediator of emotional labor strategies & burnout: a study on ASHA workers. Indian Journal of Industrial Relations. 2015 Jul 1:57-69  12. Shapira, L. B., & Mongrain, M. (2010). The benefits of self-compassion and optimism exercises for individuals vulnerable to depression. Journal of Positive Psychology, 5(5), 377–389. Crossref  13. Ivtzan, I., Niemiec, R. M., & Briscoe, C. (2016). A study investigating the effects of mindfulness-based strengths practice (MBSP) on wellbeing. International Journal of Wellbeing, 6(2), 1–13 | | | | | | | | |
| 4. | Addressing work/  workload demands | 1.Lot of work 2.Unstructured work schedule 3.Multiple engagements/  projects 4.Difficulty in managing time | Learn the techniques to efficiently plan and manage work (task) related challenges | 1. Self-Regulation, Prudence, Perseverance (endurance) 2. Wisdom/perspective, judgment (prioritizing)  3. Zest (self-motivation)  4. Open mindedness  5. Teamwork  6. Creativity  7. Social Skillfulness | 1. Planning/prioritising techniques (routine tasks, conflicts)  2. Goal-setting^14^e.g. mental contrasting  3. Improving resilience^14^e.g. resource-priming  4. Meaning: Concept of *Dharma* under a larger shared consciousness; e.g., Three Satisfying and not satisfying things (1-2/w) 5. Mindfulness: Stop/Pause | 1. Group exercises  2. Gratitude diary  3. Reflection  4. Roleplays | 1. Pen and paper  2. PowerPoint slides | Build the knowledge of techniques necessary to manage problems related to a busy and unstructured work schedule |
| 14. Niemiec RM. Character strengths interventions: A field guide for practitioners. Hogrefe Publishing; 2017 Jun 30.  15. Oettingen, G., Marquardt, M. K., & Gollwitzer, P. M. (2012). Mental contrasting turns positive feedback on creative potential into successful performance. Journal of Experimental Social Psychology, 48, 990–996  16. Stadler, G., Oettingen, G., & Gollwitzer, P. M. (2010). Intervention effects of information and self-regulation on eating fruits and vegetables over two years. Health Psychology, 29(3), 274–28  17. Fluckiger, C., Caspar, F., Grosse Holtforth, M., & Willutzki, U. (2009). Working with patients’ strengths: A micro-process approach. Psychotherapy Research, 19(2), 213–223.  18. Fluckiger, C., & Grosse Holtforth, M. (2008). Focusing the therapist’s attention on the patient’s strengths: A preliminary study to foster a mechanism of change in outpatient psychotherapy. Journal of Clinical Psychology, 64, 876–890.  19. Lai ST, O'Carroll RE. The Three Good Things'-The effects of gratitude practice on wellbeing: A randomized controlled trial. Health Psychology Update. 2017;26(1):10-8. | | | | | | | | |
| 5. | Managing conflict at individual and domestic level | 1.Physical Health Issues (tension headaches/fatigue)  2. Insufficient contribution towards domestic duties 3.Having no listening ears for expression of success and failure 4.Facing difficult situations at home due to work demands; family resistance | Learn ways by which domestic challenges in relation to work demands can be addressed or managed | 1. Forgiveness/  Self-compassion 2. Humor  3. Love  4. Kindness  5. Social intelligence  6. Perspective  7. Self-discipline/Self-control | 1. Interactions with family members (feasible?, training certificates?, ‘impact video’)  2.Social resources: Share/Vent with someone outside home  3.Positive actions towards self and others (e.g., achievements of the day, asking the other on their day)  4.Emotional regulation  5.Mindfulness^7^  e.g., ‘Three Funny Things’ activity (humor)  ‘Helping/Harming’ activity (compassion)  Managing ‘triggers’ (hot buttons)^14^ | 1. Home assignments^20^  2. Group activities  3. Impact video  4. Roleplays (positive communication)  5. Mindfulness activity | Paper and pen  PowerPoint Presentation  Supporting materials for group activities | Build the ability of the ASHA to reduce her stress due to domestic issues resulting out of work-life imbalance |
| 20. Meyers, M.C. & Meyers van. (2016). Effects of a strengths intervention on general and work-related wellbeing: The Mediating Role of Positive Affect. *J. Happiness Stud*. 18, 671-689.  21. Wellenzohn, S., Proyer, R. T., & Ruch, W. (2016a). Humor-based online positive psychology interventions: A randomized placebo-controlled long-term trial. The Journal of Positive Psychology, 11(6), 584–594.  22. Proyer, R. T., Gander, F., Wellenzohn, S., & Ruch, W. (2014a). Positive psychology interventions in people aged 50–79 years: Long-term effects of placebo-controlled online interventions on wellbeing and depression. Aging & Mental Health, 18, 997-1005.  23. Reivich, K. J., Seligman, M. E. P., & McBride, S. (2011). Master resilience training in the U. S. Army. American Psychologist, 66(1), 25–34. | | | | | | | | |
