# Supplementary material for: A character-strengths based coaching intervention to improve wellbeing of rural community health workers in Madhya Pradesh, India: Protocol for a single-blind randomized controlled trial
Source: Contemp Clin Trials Commun. 2024 Sep 27;42:101377. doi: 10.1016/j.conctc.2024.101377 (PMC11488449; doi:10.1016/j.conctc.2024.101377)
Supplement: Multimedia component 2 [file mmc2.docx]

**Modifications in the Blueprint:**

| **Modules** | **Name of the module** | **Learning Objectives** | **Methods** | **Activities** | **Strategies** |
| --- | --- | --- | --- | --- | --- |
| M-1 | Introduction to Character Strengths | To understand the scope and meanings of character strengths  To know the names of the 24-character strengths  To learn about the optimal use of character strengths  To develop the abilities to use Character Strengths | Didactic Lectures | Reflective activities, Unscripted Roleplays, Group discussions | 1. Creating a strengths habit^1^ |
| M-2 | Understanding Stressful situations | To summarize the stressful situations/problems faced by ASHAs (personal (home), professional or community (village) level).  To understand the role of the ‘self’ in these stressful situations. | Interactive discussions, Didactic lectures | Case vignettes, Reflective activities | 1. Improving Communication (Drawing from National Health Mission’s ASHA Training Module)^2^ |
| M-3 | Consequences of Stressful situations | To discuss the impact of stressful situations/problems faced by ASHAs on physical and mental health | Interactive discussions, Didactic lectures | Case vignettes, Reflective activities, Case presentations, Role-plays |  |
| M-4 | Strategies to deal with stressful situations | To identify, discuss, and learn possible strategies to deal with stressful situations. | Didactic lectures followed with ‘strategy rehearsals’ | Case presentations (Oral), Unscripted & Scripted Roleplays, Case vignettes, Group Discussions, Reflective activities, Movies/Videos | 1. Prioritization planning^3^ 2. Emotional Regulation^4^ 3. Three funny Things^1^ 4. Goal Setting using Implementation Intentions^5^ 5. Positive Actions Through Movies^1^ 6. Positive Reappraisal with Strengths 7. Three Good Things^1^ 8. Mindfulness^6^ |

**References:**

1. Niemiec, R. M. Character Strengths Interventions: A Field Guide for Practitioners. *Int. Body Psychother. J.* **16**, 84+ (2017).

2. Induction Training Module for ASHAs (National Health Mission)

3. The Eisenhower Matrix: Introduction & 3-Minute Video Tutorial. https://www.eisenhower.me/eisenhower-matrix/ (2017).

4. Gross, J. J. The Emerging Field of Emotion Regulation: An Integrative Review - James J. Gross, 1998. (1998).

5. Gollwitzer, P. M. Implementation intentions: Strong effects of simple plans. *Am. Psychol.* **54**, 493–503 (1999).

6. Kabat-Zinn, J. Mindfulness-Based Stress Reduction (MBSR) - ProQuest. (2003).
